# Concordance of the risk of neonatal respiratory morbidity assessed by quantitative ultrasound lung texture analysis in fetuses of twin pregnancies

**DOI:** 10.1038/s41598-022-13047-x

**Published:** 2022-05-30

**Authors:** Ana L. Moreno-Espinosa, Ameth Hawkins-Villarreal, Xavier P. Burgos-Artizzu, David Coronado-Gutierrez, Santiago Castelazo, Diana L. Lip-Sosa, Javiera Fuenzalida, Dahiana M. Gallo, Tatiana Peña-Ramirez, Paula Zuazagoitia, Miriam Muñoz, Mauro Parra-Cordero, Eduard Gratacòs, Montse Palacio

**Affiliations:** 1 BCNatal-Fetal Medicine Research Center (Hospital Clínic-Maternitat and Hospital Sant Joan de Déu), University of Barcelona, Institut d’Investigacions Biomèdiques August Pi I Sunyer, Carrer Sabino de Arana 1, 08028 Barcelona, Spain; 2 Centre for Biomedical Research on Rare Diseases (CIBERER), Barcelona, Spain; 3 Transmural Biotech SL, Barcelona, Spain; 4 Obstetrics Department, Hospital Santo Tomás, Universidad de Panamá (On behalf of the Iberoamerican Research Network in Obstetrics, Gynecology and Traslational Medicine), Panama City, Panama; 5 Universidad del Valle, Cali, Colombia; 6 Hospital Universitario del Valle, Evaristo García E.S.E., Cali, Colombia; 7 Hospital Clínico Universidad de Chile, Santiago de Chile, Chile

**Keywords:** Health care, Medical research

## Abstract

To evaluate the concordance of the risk of neonatal respiratory morbidity (NRM) assessed by quantitative ultrasound lung texture analysis (QuantusFLM) between twin fetuses of the same pregnancy. Prospective study conducted in twin pregnancies. Fetal ultrasound lung images were obtained at 26.0–38.6 weeks of gestation. Categorical (high or low) and continuous results of the risk of NRM were compared between twins. Fetal ultrasound lung images from 131 pairs (262 images) of twins were included. The images were classified into three gestational age ranges: Group 1 (26.0–29.6 weeks, 78 images, 39 pairs [29.8%]); Group 2 (30.0–33.6 weeks, 98 images, 49 pairs [37.4%]) and Group 3 (34.0–38.6 weeks, 86 images, 43 pairs [32.8%]). Concordance was good in Groups 1 and 3 and moderate in Group 2. In Groups 2 and 3 at least one fetus presented high-risk results in 26.5% and 11.6% of twin pairs, respectively. Only gestational age < 32 weeks, gestational diabetes mellitus, and spontaneous conception were associated with a high risk of NRM in Group 2. There was good concordance of the risk of NRM between twins < 30.0 weeks and > 34.0 weeks. From 30.0 to 33.6 weeks 26.5% of the twin pairs had discordant results, with moderate concordance of the risk of NRM.

## Introduction

Discordant neonatal respiratory morbidity (NRM), such as respiratory distress syndrome (RDS) has been described in twin pregnancies^1^. There is always clinical concern that fetal lung maturity (FLM) may differ between twins. Therefore, prediction of FLM may be helpful when premature and early term delivery of twins is expected.

The rate of pregnancy complications is higher in twin pregnancies resulting in increased neonatal morbidity linked to prematurity^2,3^. The rate of late preterm delivery may be as high as 50%^4^. The development of RDS and transient tachypnea of the newborn (TTN) are major causes of neonatal morbidity and have been described by many authors^4–7^, with several studies having evaluated the most reliable test to predict these outcomes^8–11^. Tests to estimate FLM by measuring surfactant in amniotic fluid were developed in the 1960s and 1970s after advances in the understanding of fetal lung development. However, these tests required an invasive procedure to extract and later analyze the amniotic fluid, and despite their excellent performance in determining FLM, their use has practically disappeared^12^. Indeed, an invasive procedure was a main limitation for studies in twin pregnancies using lamellar bodies, TDx-FLM, lecithin/sphingomyelin ratio or other similar tests. Therefore, information on fetal lung maturity concordance along pregnancy between co-twins is scarce, due to the invasive nature of amniocentesis, and has not been extensively studied.

In the search for a non-invasive method to obtain FLM information carried out in obstetric ultrasound in the last decade, approaches based on quantitative ultrasound analysis have specifically been explored in fetal medicine to determine the potential of ultrasound to predict FLM^13–17^.

Previous studies using quantitative ultrasound lung texture analysis (QuantusFLM)^18,19^, included a significant number of high-risk pregnancies, but the performance of this tool in particular groups of patients such as twin pregnancies has not been specifically addressed. The clinical decision to deliver twin pregnancies preterm is challenged by the presence of more than one fetus with potentially different lung maturity status.

The aim of the study was to evaluate the concordance of the risk of NRM assessed by QuantusFLM between twin fetuses of the same pregnancy at different gestational age ranges.

## Methods

### Patient recruitment and image acquisition protocol

This prospective study was performed at the Maternal–Fetal Medicine Department at the Hospital Clinic of Barcelona in collaboration with the Hospital Clínico Universidad de Chile (Chile) and Hospital Del Valle in Cali (Colombia). Eligible cases included consecutive cases of twin pregnancies between 26.0 and 38.6 weeks of gestation undergoing follow-up ultrasound during routine prenatal visits from December 2017 to March 2020. Ultrasound images were obtained following an acquisition protocol described elsewhere^19^. Briefly, a lateral axial section of the fetal thorax at the level of the 4-chamber cardiac view without any type of postprocessing manipulation was acquired, collected, and stored in the original Digital Imaging and Communication in Medicine format. The images were anonymized by removing patient information and labeled with new identification numbers and the gestational age at acquisition and later classified into three gestational age ranges: Group 1 (26.0–29.6 weeks), Group 2 (30.0–33.6 weeks) and Group 3 (34.0–38.6 weeks). The analysis included only cases in which the images of both fetuses were available in the appropriate gestational age group.

### Image processing

Images fulfilling the quality criteria were loaded via the Internet by engineers at the coordinating center through restricted access to the commercial software websitewww.quantusflm.com (www.quantusflm.org; Transmural Biotech, Barcelona, Spain) and analyzed using the new QuantusFLM version 3.0^20^. This software automatically delineates a region of interest in the fetal lung and calculates an NRM risk score (defined as the occurrence of either RDS or TTN) as a continuous variable. To evaluate the risk of NRM, continuous output NRM risk scores were binarized using the optimal cut-off point threshold computed as that maximizing accuracy in the test images, thereby obtaining a categorical result (i.e., high or low risk). The results were considered concordant when both twins were low-risk or high-risk of NRM and discordant when the risk of one twin differed from the co-twin.

### Ethical approval

All patients included in the study provided written informed consent for the use of ultrasound images and perinatal data. The study was performed in accordance with relevant guidelines and regulations and approved by the Institutional Review Board of the Hospital Clinic of Barcelona (HCB/2017/0642).

### Statistical analysis

Quantitative variables were assessed using the Shapiro–Wilk test for normality, and normally distributed variables were compared using the t-test and expressed as mean and standard deviation/standard error of the mean (SD/SEM). Non-normally distributed quantitative variables were compared using the Mann–Whitney U test and expressed as median and interquartile range (IQR: p25–75). Qualitative variables were compared using the X^2^ and Fisher exact tests. Concordance was assessed with the McNemar test in paired nominal data and intraclass correlation coefficient (ICC) with two-way mixed-effects model using absolute agreement in continuous data. We considered ICC values < 0.5, between 0.5 and 0.75, between 0.75 and 0.9, and > 0.90 as poor, moderate, good, and excellent concordance, respectively^21^.

Univariate logistic regression analysis was performed in each group to explore variables associated with high risk of NRM. A p-value < 0.05 was considered significant. Data were analyzed using STATA, v.15.0 (College Station, Texas).

## Results

### Dataset composition and baseline characteristics

**Figure 1:**
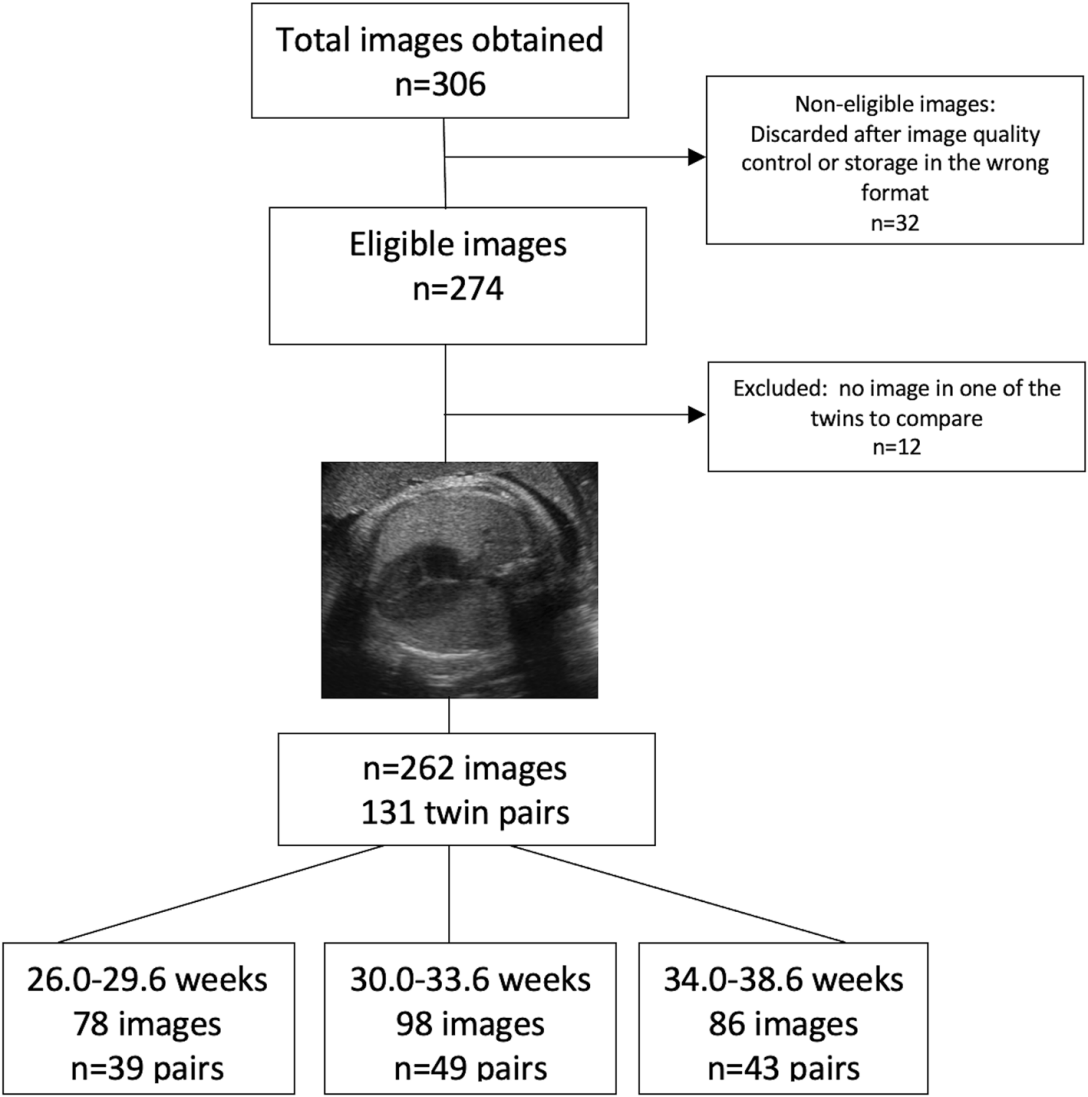
Flowchart of the eligible cases.

A total of 306 images were obtained; 32 (10.4%) were non-eligible and discarded after image quality control or having been stored in incorrect formats, and 12 (3.9%) were excluded after imaging processing due to the impossibility of obtaining an image in one of the twins to compare. The study included 131 pairs of twins (262 images). The flowchart of the eligible images is depicted in Fig. 1.

Each pair of twins contributed once and the images were classified and analyzed in three gestational age ranges: Group 1 (26.0–29.6 weeks, 78 images, 39 pairs [29.8%]); Group 2 (30.0–33.6 weeks, 98 images, 49 pairs [37.4%]) and Group 3 (34.0–38.6 weeks, 86 images, 43 pairs [32.8%]).

The baseline and clinical characteristics of the 131 women are shown in Table 1. Eighteen women (13.7%) delivered in other facilities, with perinatal results missing in 12 (9.2%).

**Table 1:** Baseline and clinical characteristics of 131 women with twin pregnancy who underwent fetal lung ultrasound examination.

Characteristic	Value
Maternal Age, years, mean (SD)	35.3 (5.7)
BMI, median (IQR)	23.0 (20.9–25.3)
Nulliparity, n (%)	90 (68.7)
**Chorionicity**	
Dichorionic, n (%)	90 (68.7)
Monochorionic, n (%)	41 (31.3)
Gestational age at delivery, mean (SD)	36.3 (2.8)
**Ethnicity**	
Caucasian	104 (79.4)
Black	1 (0.8)
Asian	2 (1.5)
Hispanic	19 (14.5)
Other	5 (3.8)
Antenatal corticosteroids, n (%)	43 (33.9)
Maternal/Fetal relevant condition, n (%)	86 (65.7)
Preeclampsia	20 (15.3)
IUGR	18 (13.7)
Diabetes	9 (6.9)
IVF	59 (45.0)
Preterm labor/PPROM	26 (19.8)

Data are presented as mean and standard deviation (SD), percentage (%), median (*IQR* interquartile range).

*BMI* body mass index, *IUGR* intrauterine growth restriction, *IVF* in vitro fertilization, *PPROM* preterm premature rupture of membranes.

### Risk for NRM and concordance between twins

**Figure 2:**
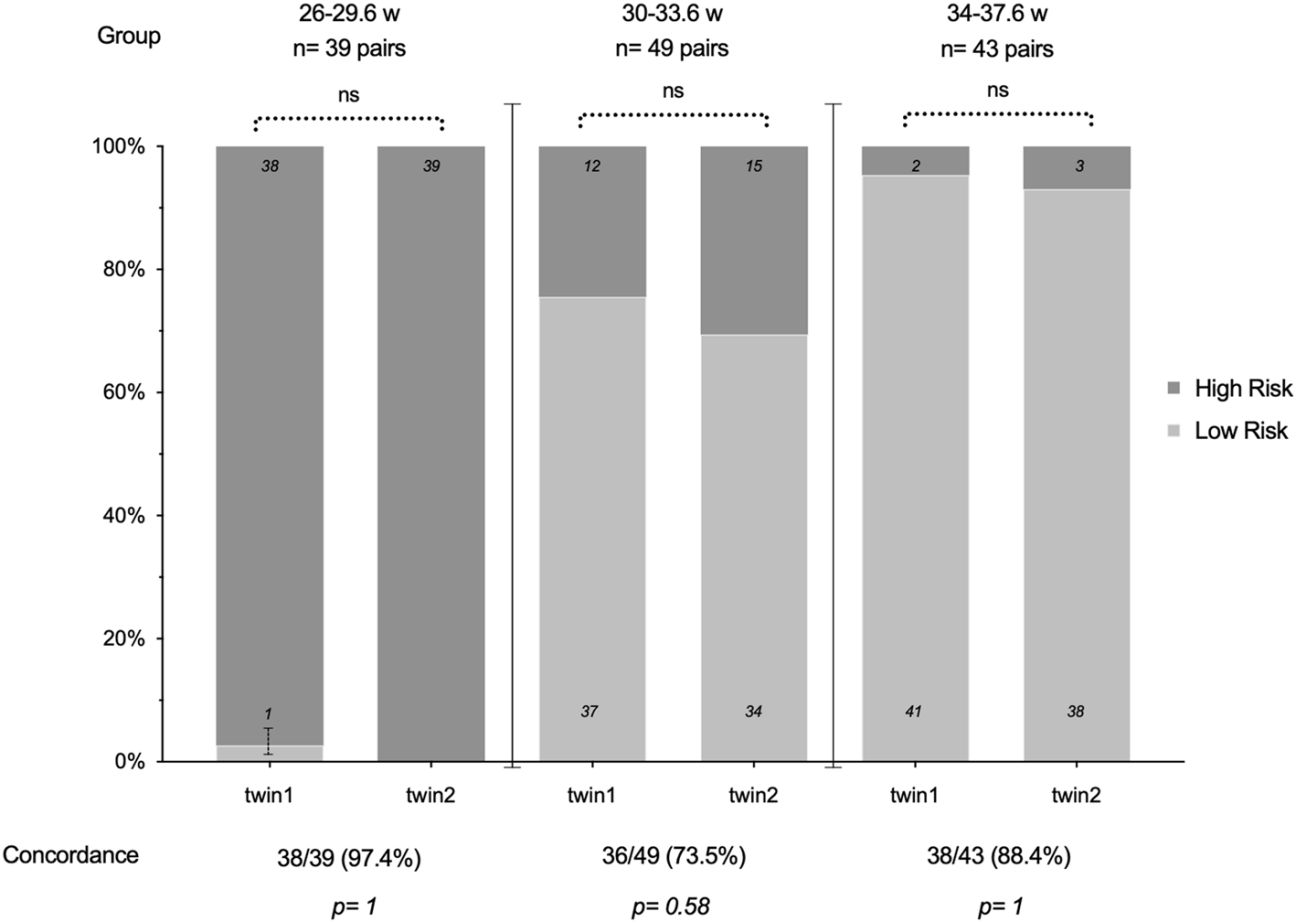
Comparison of QuantusFLM results between twins according to groups of gestational age. Data presented as frequencies or percentage (%). *p value is determined with the McNemar test of paired nominal data.

Figure 2 shows the comparison of the categorical QuantusFLM results between pairs of twins according to gestational age groups.

Group 1 (26.0–29.6 weeks) showed high risk results in all but 1 case (2.6%) achieving global concordance of 97.4% (38/39). Group 2 (30.0–33.6 weeks) presented global concordance in 73.5% (36/49) of cases. with 7 of the 36 pairs with concordant results showing high-risk results for NRM in both fetuses. Of the 13 pairs with discordant results, 61.5% (8/13) of high-risk results corresponded to the second twin. Therefore, in 20/49 (40.8%) twin pairs in Group 2, one or both fetuses had high-risk results for NRM. In group 3 (34.0–38.6 weeks) the global concordance was 88.4%; in 5/43 (11.6%) twin pairs, one fetus presented high-risk results for NRM with no cases of both fetuses scoring high-risk for NRM. In Group 1, the discordant pair of twins was dichorionic. In Group 2, 18/49 (36.7%) twin pairs were monochorionic and 31/49 (63.3%) were dichorionic. The percentage of concordant results in monochorionic (72.2% [13/18]) versus dichorionic (74.2% [23/31]) twins, was not statistically significant (p = 0.880). The discordant pairs in this group were 27.8% (5/18) monochorionic and 25.8% (8/31) dichorionic. In Group 3, 10/43 (23.3%) twin pairs were monochorionic and 33/43 (76.7%) were dichorionic. The percentage of concordant results in monochorionic (90.0% [9/10]) versus dichorionic (87.9% [29/33]) twins, was not statistically significant (p = 0.855). The discordant pairs were 10.0% (1/10) monochorionic and 12.1% (4/33) dichorionic.

The analysis for continuous QuantusFLM results showed good concordance in Groups 1 and 3, [ICC = 0.781 (95% CI 0.573–0.88; p ≤ 0.001)] and [ICC = 0.900 (95% CI 0.817–0.946; p ≤ 0.001)], respectively; while concordance was moderate in Group 2 [ICC = 0.522 (95% CI 0.156–0.730; p = 0.006)].

The mean gestational age at fetal lung ultrasound examination was lower for high-risk vs. low-risk results in all groups; Group 1 (27.5 vs. 29.4 weeks); Group 2 (31.2 vs. 32.0 weeks) and Group 3 (34.8 vs. 35.4 weeks), being statistically significant only in Group 2 (p ≤ 0.001).

### Univariate analysis

**Figure 3:**
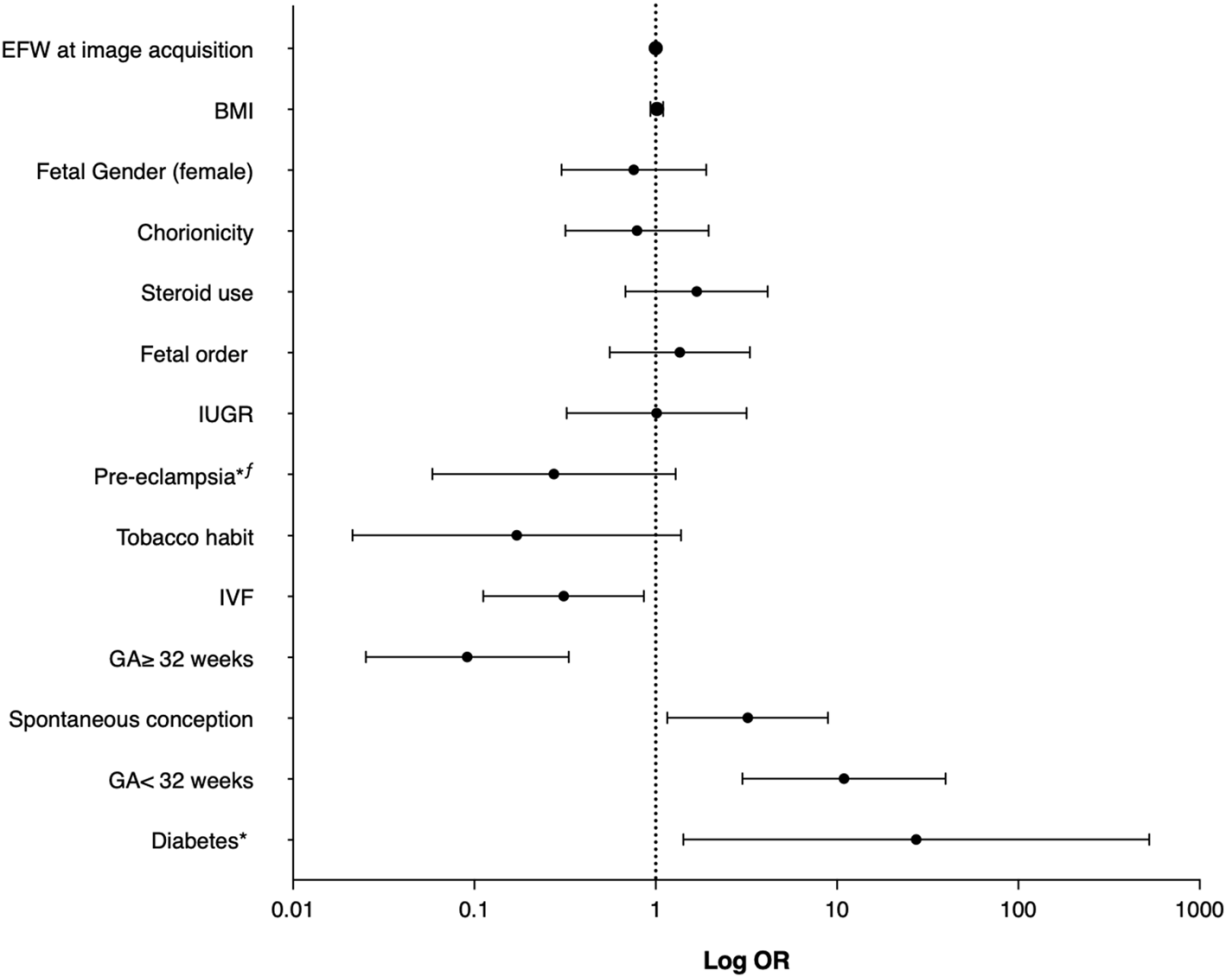
Individual risk for increased risk of neonatal respiratory morbidity according to QuantusFLM results in Group 2 (30.0–33.6 weeks). All p values and confidence intervals were calculated with robust bias-corrected logistic regression. *Adjusted for gestational age at the time of image acquisition. ^ƒ^Adjusted for steroid use. *EFW* estimated fetal weight, *BMI* body mass index, *IUGR* intrauterine growth restriction, *IVF* in vitro fertilization, *GA* gestational age.

Other variables were evaluated to determine the characteristics potentially associated with high-risk NRM results. Figure 3 depicts the individual risk for high-risk of NRM according to the QuantusFLM results in Group 2. In this group, spontaneous conception, GA < 32 weeks and diabetes were significantly associated with high-risk results. Regarding the mode of conception, images with high-risk results from fetuses conceived by IVF (6 images) were obtained at a mean GA age of 31.9 weeks, compared to high-risk results from images of spontaneously conceived fetuses (21 images) that were obtained at a mean GA of 30.9 weeks. Regarding diabetes, images with high-risk results (4 images) were obtained at a mean GA of 31.4 weeks. Supplementary Figs. S1 and S2 show the individual risk of high-risk of NMR according to QuantusFLM results in Groups 1 and 3, respectively.

### Perinatal and neonatal results

Table 2 shows the perinatal and neonatal results of the 238 newborns enrolled in the study. The prevalence of NRM was 14.3% (33/231), of which 72.7% (24/33) were diagnosed as TTN and 27.3% (9/33) as RDS. All newborns diagnosed with RDS received at least one type of respiratory support (oxygen ≥ 40%, continuous positive airway pressure (CPAP), non-invasive/bilevel positive airway pressure, intubation, high frequency ventilation, and use of surfactant). Of the newborns diagnosed with TTN, 83.3% (20/24) received CPAP. Male newborns presented a higher prevalence of NRM (69.7% [23/33]) and NRM was equally distributed among first 16/33 (48.4%) and second 17/33 (51.6%) twins.

**Table 2:** Perinatal and neonatal outcomes of the newborns included in the study.

Characteristics	First twin n = 131	Second twin n = 130^b^	p value
**Mode of delivery, n (%)**^**a**^			0.874
Spontaneous vaginal delivery	31 (26.1)	27 (22.7)	
Operative vaginal delivery	3 (2.5)	3 (2.5)	
Non-elective cesarean section	17 (14.3)	21 (17.7)	
Elective cesarean section	68 (57.1)	68 (57.1)	
Birthweight, mean (SD)	2335 (408.0)	2255 (513.7)	0.184
Female gender, n (%)	62 (52.1)	61 (51.3)	0.897
Apgar at 5 min < 7, n (%)^a^	0 (0)	0 (0)	1
pH UA < 7.10, n (%)^a^	3/110 (2.7)	8/107 (7.5)	0.111
Phototherapy-Hyperbilirubinemia, n (%)^a^	12/116 (10.3)	19/115 (16.5)	0.168
NICU admission, n (%)^a^	28 (24.1)	30 (26.1)	0.733
Length of stay at NICU, mean (SD)^a^	15.4 (13.3)	18.7 (37.1)	0.658
Need for respiratory support (any), n (%)	15 (12.9)	13 (11.3)	0.705
Oxygen therapy ≥ 40%, n (%)	4 (3.5)	4 (3.5)	0.990
CPAP, n (%)	15 (12.9)	12 (10.4)	0.555
NIV/BIPAP, n (%)	2 (1.7)	2 (1.7)	0.993
Intubation required, n (%)	2 (1.7)	1 (0.9)	0.566
High frequency ventilation (HFV), n (%)	0 (0)	1 (0.9)	0.314
Surfactant use, n (%)	2 (1.7)	1 (0.9)	0.566
**Neonatal respiratory morbidity, n (%)**	16 (13.8)	17 (14.8)	0.830
Transient tachypnea of the newborn	13 (11.2)	11 (9.6)	0.683
Respiratory distress Syndrome	4 (3.5)	5 (4.4)	0.724

Data are presented as mean and standard deviation (SD), percentage (%).

*UA* umbilical artery, *NICU* neonatal intensive care unit, *CPAP* continuous positive airway pressure, *NIV/BIPAP* non-invasive ventilation/Bi-level positive airway pressure.

^a^Variables with missing values.

^b^There was an intrauterine fetal demise in one dichorionic twin pregnancy at 35 weeks of gestation.

## Discussion

We prospectively evaluated for the first time the concordance of the risk of NRM between twins with non-invasive quantitative ultrasound lung texture analysis, obtaining 97.4% (Group 1), 73.5% (Group 2) and 88.4% (Group 3) concordance in the risk of NRM. Indeed, the risk of NRM was discordant between twin pairs in 2.5% (1/39), 26.5% (13/49) and 11.6% (5/43) in Groups 1, 2 and 3, respectively. We found 97.4% (77/78) of fetuses with high-risk results in Group 1, 27.6% (27/98) in Group 2 and 5.8% (5/86) in Group 3. These results achieved good concordance in Groups 1 and 3, being moderate in Group 2. Knowing the status of fetal lung maturity may be helpful in clinical practice since frequent obstetric complications impel delivery planning in twin pregnancies in the late-preterm and early-term weeks of gestation. In such circumstances delaying delivery could be ambiguous, and information of lung maturity may help to weigh maternal and fetal risks. Our univariate logistic regression analysis showed that spontaneous conception, gestational diabetes mellitus and gestational age < 32 weeks were associated with a high-risk of NRM in Group 2. Variables associated with high-risk results were not found in Groups 1 or 3.

A decade ago, the prediction of fetal lung maturity relied almost solely on laboratory tests of amniotic fluid obtained invasively (amniocentesis). Previous studies using these tests have described similar differences in FLM to those of our study at a given gestational age and many gestational factors had been proposed as predictors of FLM concordance.

Regarding gestational age and chorionicity, Mackenzie performed amniocentesis in 92 pairs of diamniotic twins and found that at extremes of gestational age (28, 29, 36 and 37 weeks), twins were 100% concordant in lung status, while lung concordance was significantly lower in twins of 33–35.9 weeks, and even lower at 30–32.9 weeks. The influence of chorionicity, estimated fetal weight discordance and fetal gender concordance were not predictive of lung maturity^22^. In a study including 42 pairs of twins, Leveno et al. reported a high correlation between lecithin/sphingomyelin (L/S) ratios between twins A and B which was not related to birth order, infant sex, birthweight discordance, or zygosity^23^. Following amniocentesis in 47 twin pregnancies (26–36 weeks), Winn et.al, reported that FLM was biochemically comparable between twins A and B^24^, being in line with our results showing concordant results in 97.4%, 73.5% and 88.4% of twins in Groups 1, 2 and 3, respectively. Similar to our results, McElrath, et al. found that after 31.0 weeks, twin gestations had significantly higher levels of TDx (surfactant/albumin ratio assay) of FLM values^25^. Another study by Whitworth et al. showed that when L/S ratios were stratified by gestational age at amniocentesis, the mean percentage difference of the ratios was 25% at ≤ 32 weeks and 16% at > 32 weeks. In all the sets of twins, in which the ratios were discordant, the gestational age at amniocentesis was ≤ 32 weeks^26^. Dobbie et al. explored the correlation of biochemical ratios [L/S, phosphatidyl glycerol/sphingomyelin (PG/S) and phosphatidyl inositol/sphingomyelin (PI/S)] of FLM tests in 32 pairs of twins. They found a weak correlation for L/S ratio but a much-improved correlation for PG/S and PI/S. Concordance was greater in monochorionic pregnancies^1^. In a large study of 132 dichorionic and 125 monochorionic twins, Tsuda et al. examined the amniotic lamellar body count (LBC) and found that the concordance of the LBC results for the cut- off value was also significantly lower in dichorionic than in monochorionic twins^27^.

We found no association of fetal gender with the QuantusFLM results, but we did not test the results with the outcome at birth. However, the presence of RDS at birth was more prevalent in male newborns, similar to the studies by Mulla et al.^28^ and Steen et al.^29^.

Maternal diabetes has commonly been reported as a factor related to FLM^30,31^ and FLM tests^32,33^. It has also been associated with mixed results such as more frequent admission to neonatal intensive care units and greater birthweight but not with greater respiratory morbidity in twin pregnancies^34,35^. In our study gestational diabetes was a statistically significant predictor of high-risk results in Group 2; however, the number of cases was limited. This can be expected in these weeks of transition from pulmonary immaturity to maturity, since this association was not found in Group 3.

Assisted reproductive techniques have been associated with adverse perinatal outcomes in twin pregnancies^36–40^. Literature correlating fetal lung maturity and ART is scant. Tsuda et al., evaluated the impact of fertility treatment on NRM in twin pregnancies using amniotic LBC and found no statistically significant associations between fertility treatments and the rates of RDS/TTN^41^. We found a statistically significant association of IVF treatment to low-risk results in Group 2. When the analysis was performed by gestational age, we found that images with high-risk results from fetuses conceived by IVF were obtained at a mean gestational age of 31.9 weeks, compared to high-risk images of spontaneously conceived fetuses that were obtained at a mean gestational age of 30.9 weeks. This finding may explain why IVF has been found to be a protective factor in this group.

The aim of the present study was not to test performance but rather to explore the concordance of the high- or low-risk of NRM in prospectively followed twin pairs. This study showed a relevant number of cases of fetal lung immaturity, at least in one of the fetuses, in the gestational age ranges of Groups 2 and 3, which should be considered if delivery is expected. While in Group 2, 59.2% of pairs were already at low-risk, 11.6% in Group 3 remained at high-risk, in line with studies in which FLM was evaluated by invasive techniques.

The main strength of this study is first, the fact that a non-invasive technique was used to evaluate the individualized risk for NRM for each twin; and secondly, our study included a larger number of pairs than the studies by Leveno et al.^23^, Mackenzie^22^ and Winn et al.^24^, who included no more than 92 pairs of twins. However, some limitations are of note: respiratory morbidity was not clinically addressed immediately after the test. However, the study was designed to evaluate the concordance of the test in utero between twins, and this may help in the clinical decision-making process. Questions about a possible more advanced or delayed lung maturation process throughout pregnancy have been raised for years regarding fetuses with conditions such as growth restriction or maternal diabetes. However, information on this topic is scarce due to the limitation of the invasive nature of amniocentesis. The data obtained in this study show that discordant lung maturation could be expected in different ranges of gestational age, and this should be taken as a proof of concept.

Indeed, a multicenter study demonstrated that QuantusFLM has an accuracy of 86.5%, like that of invasive techniques, although a further study to evaluate the performance of this technique in twins is forthcoming. This information will be decisive to assess whether the tool could be added to the management of twins in clinical protocols or guidelines. In the meantime, while the information shown in this study may not by itself be decisive when planning delivery, knowledge of the probability of discordance in a particular range of gestational age can help. Indeed, this could be relevant where a neonatal unit is not available.

In conclusion, there was good concordance of the risk of NRM between twins < 30.0 weeks and > 34.0 weeks. From 30.0 to 33.6 weeks 26.5% of the twin pairs had discordant results. Gestational age < 32 weeks, gestational diabetes mellitus and spontaneous conception were found to be associated to high-risk results of NRM in this group. In 11.6% of pregnancies in the group above 34 weeks, at least one fetus of these pairs, showed high-risk of NRM. This information may be helpful to plan and guide obstetricians in the clinical decision-making process.

## Supplementary Information

Supplementary Figures.

## Data Availability

The datasets generated and/or analyzed during the current study are not publicly available due to restrictions according to patient privacy regulations but are available from the corresponding author on reasonable request.
