# Supplementary material for: Concordance of the risk of neonatal respiratory morbidity assessed by quantitative ultrasound lung texture analysis in fetuses of twin pregnancies
Source: Sci Rep. 2022 May 30;12:9016. doi: 10.1038/s41598-022-13047-x (PMC9151662; doi:10.1038/s41598-022-13047-x)
Supplement: Supplementary file 1 — Supplementary Figures. [file 41598_2022_13047_MOESM1_ESM.pdf]

Concordance of the risk of neonatal respiratory morbidity assessed by quantitative ultrasound lung texture analysis in fetuses of twin pregnancies

*Ana L. Moreno-Espinosa[1,4], Ameth Hawkins-Villarreal[1,4], Xavier P. Burgos-Artizzu[1,3], David Coronado-Gutierrez[1,3], Santiago Castelazo[1], Diana L. Lip-Sosa[1], Javiera Fuenzalida[1], Dahiana M. Gallo[5,6], Tatiana Peña-Ramirez[5,6], Paula Zuazagoitia[7], Miriam Muñoz[1], Mauro Parra-Cordero[7], Eduard Gratacòs[1,2] and Montse Palacio[1,2].

1- BCNatal - Fetal Medicine Research Center, (Hospital Clínic and Hospital Sant Joan de Déu), University of Barcelona, Institut d'Investigacions Biomèdiques August Pi i Sunyer, Barcelona, Spain.
2- Centre for Biomedical Research on Rare Diseases (CIBERER), Barcelona, Spain.
3- Transmural Biotech SL, Barcelona, Spain.
4- Obstetrics Department, Hospital Santo Tomás, Universidad de Panamá, Panamá City, Panamá. On behalf of the Iberoamerican Research Network in Obstetrics, Gynecology and Traslational Medicine.
5- Universidad del Valle, Cali, Colombia.
6- Hospital Universitario del Valle, Evaristo García E.S.E., Cali, Colombia
7- Hospital Clínico Universidad de Chile, Santiago de Chile, Chile.

SUPPLEMENTARY INFORMATION

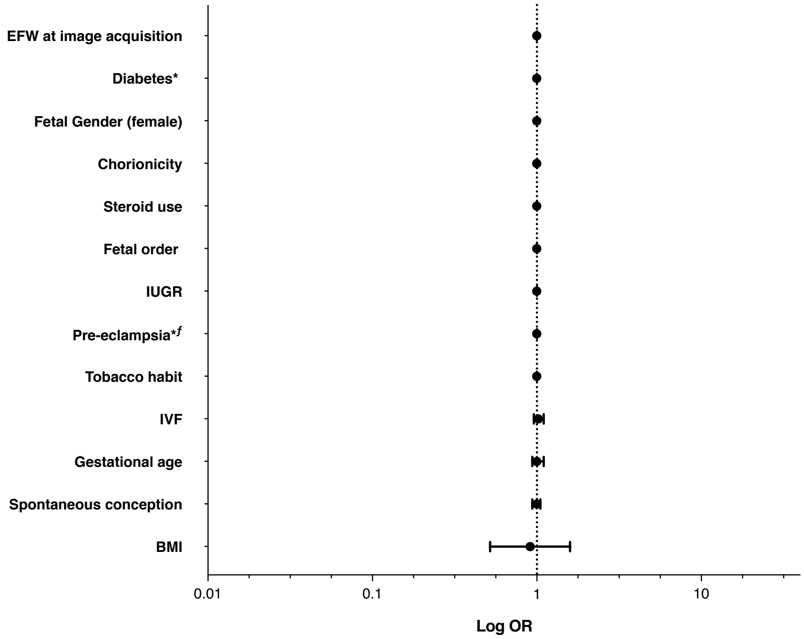

Supplementary Figure S1. Individual risk for increased risk of neonatal respiratory morbidity according to QuantusFLM® results in Group 1 (26.0 – 29.6 weeks). All *p* values and confidence intervals were calculated with robust bias-corrected logistic regression. *Adjusted for gestational age at the time of image acquisition. [f] Adjusted for steroid use. EFW: estimated fetal weight, BMI: body mass index, IUGR: intrauterine growth restriction, IVF: *in vitro* fertilization, GA: gestational age.

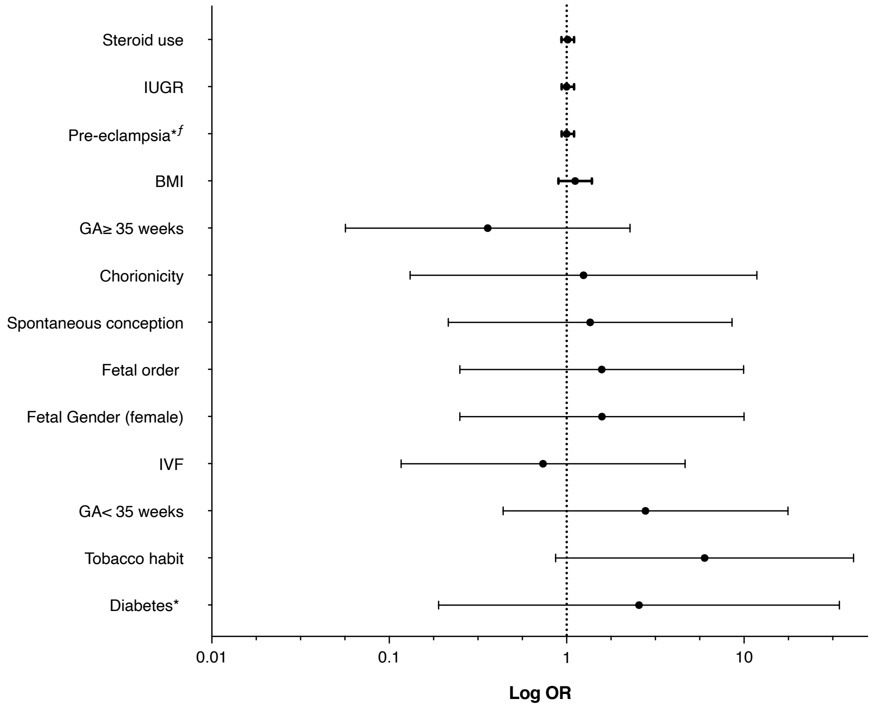

Supplementary Figure S2. Individual risk for increased risk of neonatal respiratory morbidity according to QuantusFLM® results in Group 3 (34.0 – 38.6 weeks). All *p* values and confidence intervals were calculated with robust bias-corrected logistic regression. *Adjusted for gestational age at the time of image acquisition. ^{f} Adjusted for steroid use. EFW: estimated fetal weight, BMI: body mass index, IUGR: intrauterine growth restriction, IVF: *in vitro* fertilization, GA: gestational age.
